# Analysis of the repeatability of the exhaust pollutants emission research results for cold and hot starts under controlled driving cycle conditions

**DOI:** 10.1007/s11356-018-1983-5

**Published:** 2018-04-20

**Authors:** Artur Jaworski, Hubert Kuszewski, Adam Ustrzycki, Krzysztof Balawender, Kazimierz Lejda, Paweł Woś

**Affiliations:** Faculty of Mechanical Engineering and Aeronautics, Department of Combustion Engines and Transport, Rzeszow University of Technology, 8 Powstancow Warszawy Ave, 35-959 Rzeszow, Poland

**Keywords:** Vehicle emissions, Exhaust measurements, Repeatability, Cold start test, Hot start test, Chassis dynamometer testing

## Abstract

Measurement of car engines exhaust pollutants emissions is very important because of their harmful effects on the environment. This article presents the assessment of repeatability of the passenger car engine exhaust pollutants emission research results obtained in the conditions of a chassis dynamometer. The research was conducted in a climate chamber, enabling the temperature conditions to be determined from − 20 to + 30 °C. The emission of CO, CH_4_, CO_2_, NO_X_, THC, and NMHC was subjected to the analysis. The aim of the research is to draw attention to the accuracy of the pollutant emission research results in driving cycles, and the comparison of pollutant emission results and their repeatability obtained in successive NEDC cycles under cold and hot start conditions. The results of the analysis show that, in the case of a small number of measurements, the results repeatability analysis is necessary for a proper interpretation of the pollutant emission results on the basis of the mean value. According to the authors’ judgment, it is beneficial to determine the coefficient of variation for a more complete assessment of exhaust emission result repeatability obtained from a small number of measurements. This parameter is rarely presented by the authors of papers on exhaust components emission research.

## Introduction

The harmful effects of transport on the environment are mainly related to the emission of gaseous and particulate pollutants by internal combustion engines. For many years, work has been conducted to minimize energy consumption and reduce exhaust pollutants emissions. This also applies to the introduced emission standards and testing procedures for internal combustion engines. In the case of passenger cars and light commercial vehicles, Euro procedures, which include standards for exhaust pollutants emissions during driving cycles, under controlled conditions, on a chassis dynamometer, and road tests apply in the European Union (European Commission E 2007, 2015, 2017).

Limited reserves of crude oil, which is the primary raw material for the production of gasoline and diesel, imply the search for replacement fuels. Their introduction should not result in an increase in exhaust pollutants emissions above the limit levels specified in the standards.

The emissions researches conducted by different laboratories show large differences in the values of the research results. This is due to the very high number of factors that affect the exhaust components emission levels (Joumard et al. 2013). Those are the factors related to the research equipment used, the object of the research and their conditions. The main factor on which the pollutant emissions level depends is the driving cycle. Currently, new models of cars should go through legislative research in accordance with WLTP (World Harmonized Light Vehicle Research Procedure), in which WLTC cycles (World Harmonized Light Vehicles Test Cycle) replace the NEDC test (European Commission E 2017; Heinfellner et al. 2016). More emphasis is placed on the tests that involve the cycles that are more closely related to real-time driving cycles present on the roads (Pielecha et al. 2016; Merkisz and Rymaniak 2017), reproduced on the dynamometer bench, or the conduct of research organized on the road (RDE—Real Driving Emissions). However, there are divergent data on the emission levels differences derived from NEDC and WLTC tests. According to papers (Pelkmans and Debal 2006; May et al. 2014), the emission of pollutants in real traffic conditions is often higher than for NEDC. Also in WLTP-compliant tests (Marotta et al. 2015), higher emission factors were reported for the entire test compared with NEDC for CO and NO_X_ in the case of gasoline engines, and for NO_X_ in the case of diesel engines. On the other hand, in paper (Mast 2014), NO_X_ emission in the case of a petrol engine was lower for the entire WLTC test than for NEDC. Comparative tests of the exhaust pollutants emissions for NEDC and WLTC tests are also the object of numerous papers (Andersson et al. 2014; May et al. 2014; Bielaczyc et al. 2014, 2015; Ligterink et al. 2016). However, it is difficult to compare the emission results obtained from the tests with significantly different parameters.

A difficulty with regard to the research testing is still the question of determining the distribution of the research results. For the tests conducted on public roads, in real traffic conditions, it is difficult to talk about high repeatability. Therefore, tests on dynamometers, including those based on NEDC tests, remain favorable in relation to such R&D tests, for which their differences of results are not large.

Because in many cases the influence of introduced structure and operating changes on fuel consumption and emission of engine exhaust pollutants is low (Concave 2016; Martini et al. 2013), it is important to determine the accuracy of the conducted researches. Pollution emissions tests conducted on the chassis dynamometer are a very complex emission. The test results depend, among other things, on the reproducibility of the driving cycle conducted by the car driver, the climatic conditions, the physicochemical properties of the fuel, the engine and vehicle design and operating parameters, and the accuracy of the used test equipment. A broad analysis of the influence of the selected factors on the pollutants emission in the research tests is included in the paper of Joumard et al. (2013).

The pollution emissions research conducted in road conditions or on the chassis dynamometer are very expensive, which is connected mainly with the high costs of the test equipment, the costs of electricity, and working gases. Therefore, the question of the minimum number of measurements that must be conducted to make the test result acceptable, arises. In the case of approval tests, according to the regulations (European Commission E 2007, 2015, 2017), the number of emission measurements for a type 1 test, depending on the results obtained, may be one, two, or three. For this reason, it is very important for the accuracy and repeatability of the measurements that they be performed under repeatable conditions, which include:

- Use of the same test method
- Tests in the same laboratory
- Tests conducted by the same operator
- Tests with the same equipment
- Short-term tests

The engine’s thermal state is a factor that has a significant influence on the exhaust pollutants emission test results (Bielaczyc et al. 2001; Kwak et al. 2007; Kan et al. 2017). It involves both the engine control strategy (including fuel injection, ignition) and the catalytic converter efficiency. Such a large number of factors, each of which has a specific range of values, results in variability of results. Therefore, it is important to determine the extent of repeatability of the results for a given research method when conducting the research to determine the effect of design or operational changes on the exhaust pollutants emission. The accuracy of the pollutant emission research results is the object of many paper, e.g. (Myung et al. 2009; Chłopek and Rostkowski 2015; Chłopek and Szczepański 2015; Giechaskiel et al. 2008; Joumard et al. 2009, 2013; Suarez-Bertoa et al. 2014, 2015; Balawender et al. 2016; McCormick et al. 2006; Kim and Lee 2011).

Because the highest harmfulness of exhaust pollutants occurs in areas of densely populated cities, particular attention should be paid to the emission of toxic compounds in the urban part of the driving cycles and the evaluation of the accuracy of the results obtained.

This paper presents the analysis of the repeatability of the results for the exhaust pollutants emission test in a passenger car engine, powered by gasoline, according to the NEDC procedure. The tests were conducted from the cold and hot starts of the engine. The aim of the research is to emphasize the importance of repeatability of the exhaust pollutants emission tests, in particular for a small number of measurements. Attention has also been drawn to the often overlooked coefficient of variation (CV), which allows for a more accurate assessment of the test results. The authors believe that determination of the coefficient of variation should be helpful in assessing the reproducibility of results obtained by various laboratories. There are currently no standards for specifying a satisfactory value of variation coefficients regarding exhaust emission research in stationary tests of motor vehicles.

## Experimental methodology

### Experimental equipment

**Fig. 1:**
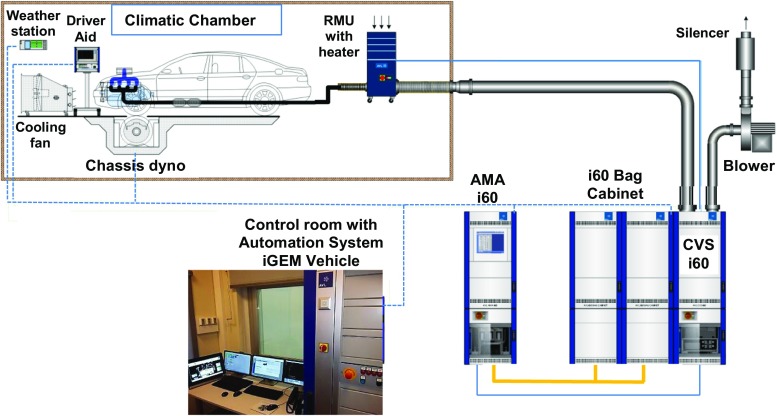
Climatic chassis dyno exhaust measurement system

The research bench is located in the Laboratory of Automotive Ecology at the Faculty of Mechanical Engineering and Aeronautics, of the Rzeszow University of Technology (Fig. 1). The Department of Combustion Engines and Transport of the Rzeszow University of Technology has worked for many years now to reduce the harmful effects of transport on the environment. This concerns the improvement of the internal combustion engines and vehicles design, and the search for alternative fuels (Kuszewski et al. 2017a, b). At present, exhaust pollutants emission testing for passenger car engines is conducted. The tests are conducted on the engine dynamometer, in real road conditions and under controlled climatic conditions according to the European NEDC driving cycle.

The tests concerning the paper in question were conducted on the chassis dynamometer Zoellner ROADSIM 48 manufactured by AVL built in a climate chamber. The basic specifications of the chassis dynamometer are provided in Table 1.

**Table 1:** Basic technical data of AVL ROADSIM 48″ chassis dynamometer (AVL 2014)

Analyzed quantity	Value
Rated power	153 kW
Instantaneous power	258 kW
Maximum speed	200 km/h
Maximum continuous tractive force	5987 N
Maximum instantaneous tractive force	10,096 N
Tractive force measurement error	≤0.1%
Speed measurement error	≤0.02 km/h
Distance measurement error	0.001%/m

The AVL climate chamber enables temperatures between − 20 and + 30 °C. The measurement bench includes the AVL CVS i60 exhaust dilution system and the AVL AMA i60 exhaust analysis system. The basic data of those systems are summarized in Tables 2 and 3.

**Table 2:** Specification of used AVL CVS i60 GD system (AVL 2010)

Parameter	CVS i60 GD
Flow rate	
Diluted exhaust	max 30 m^3^/min
Bag fill rate	≈ 4;6;10 l/min
Absolute pressure sensor measurement range	700–1100 hPa
Absolute pressure sensor measurement quality	± 0.25% of the set range
Difference pressure sensor measurement range	0–200 hPa
Temperature sensor measurement range	273–373 K
Temperature sensor measurement quality	± 1 K
Humidity sensor measurement range	0–100% rel. air humidity

**Table 3:** Specification of used AMA i60 analysers (AVL 2013)

Parameter\analyzer	CLD i60 LD	FID i60 LCD	IRD i60 CO_2_ L	IRD i60 L
Measured components	NO and NO_X_	THC and CH_4_	CO_2_	CO
Noise	≤ 1% of range full scale	≤ 0.5% of range full scale	≤ 1% of range full scale	≤ 1% of range full scale
Drift	≤ 1% of range full scale/24 h	≤ % of range full scale/24 h	≤ 1% of range full scale/24 h	≤ 1% of range full scale/24 h
Reproducibility	≤ 0.5% of range full scale	≤ 0.5% of range full scale	≤ 0.5% of range full scale	≤ 0.5% of range full scale
Linearity	≤ 2% of measured value (10–100% of range full scale) ≤ 1% of range full scale whichever is smaller	≤ 2% of measured value (10–100% of range full scale) ≤ 1% of range full scale whichever is smaller	≤ 2% of measured value (10–100% of range full scale) ≤ 1% of range full scale whichever is smaller	≤ 2% of measured value (10–100% of range full scale) ≤ 1% of range full scale whichever is smaller

The object of the test was a passenger car manufactured in 2003 with a mileage of ≈ 60,000 km, whose basic specifications are shown in Table 4. To obtain the simulated road load maximally close to the resistance of the real road, coast-down tests have been conducted, which were implemented in the speed range from 130 km/h to zero. A non-contact system with optical sensor DATRON DLS-2 was used for the measurement of the coast-down parameters. Based on the determined coast-down times, the following equation of the road load was determined:

1 $$ {F}_0=155.11-1.2343\kern0.5em V+0.4685\kern0.5em {V}^2 $$

where *F*_*o*_ is the road load (*N*) and *V* is the vehicle speed (km/h).

**Table 4:** Technical data of the tested vehicle

Parameter	Value
Length/width/height	4665/1760/1445 mm
Wheelbase	2670 mm
Weight	1430 kg
Engine type	Petrol (gasoline)
Fuel System	Multi-point injection
Engine displacement	1998 cm^3^
Engine power	115 kW @ 6000 rpm
Engine torque	190 N m @ 4500 rpm
Number of cylinders	4
Number of valves	16
Emission standard	Euro 4
Wheel drive	Front
Number of gears (manual transmission)	5
Tyre size	205/55 R16

**Fig. 2:**
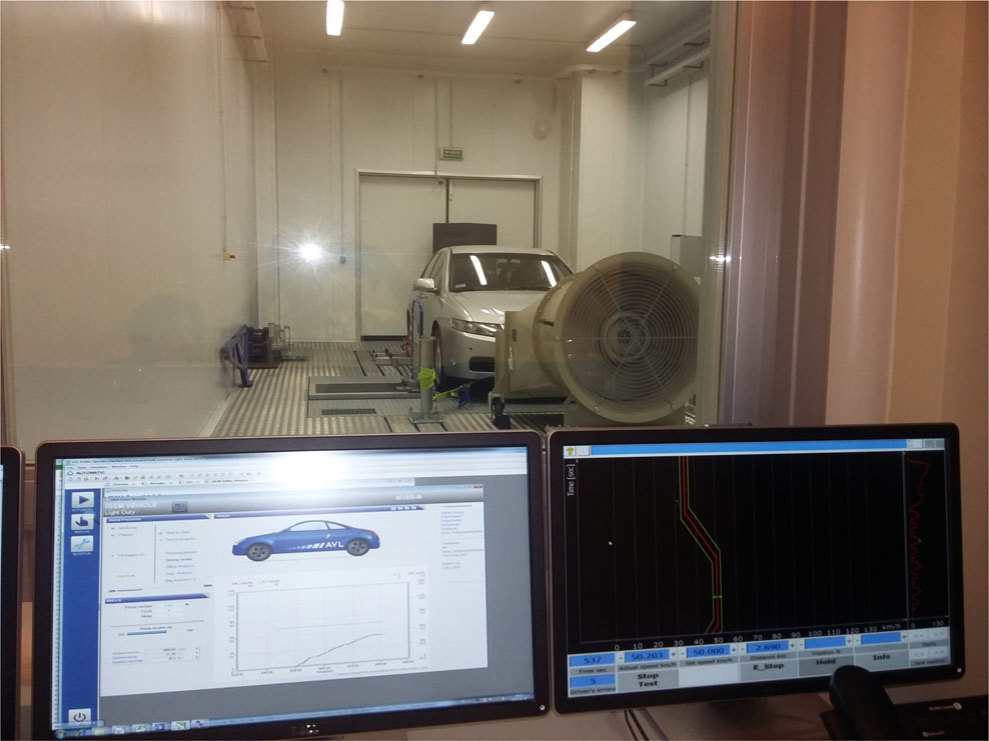
A view of the car on the research set-up

View of the tested car on the research set-up is shown in Fig. 2.

### Research methodology

**Fig. 3:**
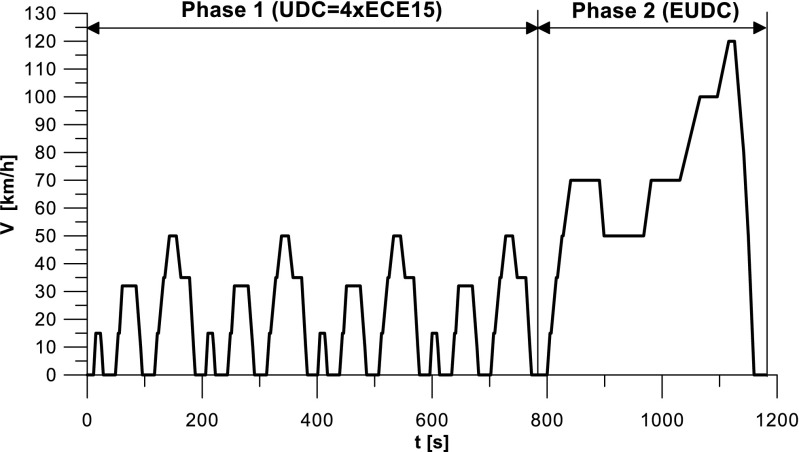
NEDC test cycle

The tests were performed according to the NEDC test procedure, which consists of two phases: UDC and EUDC (Fig. 3). The tests were performed from a cold start and hot start. Before cold start measurements, the car was conditioned for a period of 6 to 12 h in a climate chamber of 21 ± 1 °C and relative humidity of 40 ± 1%. In the case of hot start tests, the engine coolant temperature before the test was 90 ± 2 °C.

The exhaust pollutants emission measurements were conducted with a constant volume sampling system (CVS i60). Prior to each test, the exhaust analysis system was calibrated. The air diluted exhaust was directed during the bag analysis cycle and the modal analysis, i.e., measurement of diluted exhaust concentrations, was performed in parallel using an AVL AMA i60 exhaust analysis system. After the test completion, the pollutant emissions average values, for the below exhaust components were calculated in individual phases (UDC, EUDC, NEDC): THC, CH_4_, NMHC, NO_X_, CO, CO_2_. The tests were conducted using the AVL PUMA Open system with iGEM Vehicle software and with automatic exhaust emissions measurement.

2After a series of three cold start tests (tests 1–3) and three hot start tests (tests 4–6), the measurement method accuracy was analyzed and the repeatability of the test results was assessed.

The precision is defined as the degree of compatibility between independent test results obtained under the established conditions (PN-ISO 5725-1 2002; PN-ISO 5725-2, 2002). The precision depends only on the distribution of random errors and there is no reference to the real value. Normally, the standard deviations of the test results, the values of which are the smaller the higher is the precision, constitute a measure for the precision.

Repeatability is the precision when the repeatability conditions are met. To evaluate the repeatability, the following parameters were determined:

- Measurement results average values *x*_av_
- Minimum values *x*_min_ and maximum values *x*_max_
- Range *R*_x_
- Standard deviation of repeatability *s*_r_
- Repeatability limit *r*
- Coefficient of variation CV

The standard deviation was accepted as a measure of the test results dispersion, which was calculated as follows:

2 $$ {s}_r={\left(\frac{\sum \limits_{\mathrm{i}=1}^{\mathrm{n}}{\left({x}_{\mathrm{i}}-{x}_{av}\right)}^2}{n-1}\right)}^{1/2} $$

where *s*_r_ is the standard deviation of repeatability, *x*_*i*_ is the result of the *i*-th measurement, *x*_av_ is the arithmetic mean of the results, and *n* is the number of measurements.

The repeatability limit *r* is defined as a value which, with a probability of 95%, is not exceeded by the absolute value of the difference between the two tests results obtained when the repeatability conditions are met.

In the case of standardized methods, in normal laboratory practice, at least two parallel designations are executed, and the absolute difference between the results is compared with a certain critical value, which is the repeatability limit determined by the following formula (PN-ISO 5725-6 2002):

3 $$ r=2.8\cdot {s}_r, $$

where *s*_r_ is the standard deviation of repeatability indicated for all the measurements.

It is recommended that the absolute difference of the measurement results does not exceed the repeatability limit. If this value does not exceed *r*, then each result is considered to be acceptable and their mean is given as the final result.

The relative value that was adopted to assess the unrepeatability of the measurement results of particular pollutants is the CV, calculated from the following formula:

4 $$ \mathrm{CV}=\frac{s_r}{x_{\mathrm{av}}}\cdot 100. $$

The above measures were set for all emissions indicators of the analyzed exhaust components, both for the entire test as well as for the individual phases (UDC, EUDC).

## Results and discussion

The results for the emission of toxic pollutants in exhaust obtained in subsequent tests according to the NEDC test are presented in Table 5 (for cold start tests) and in Table 6 (for hot start tests). As the cold start test was conducted in accordance with the approved procedure, Euro 4 emission limit values are shown in Table 5.

**Table 5:** Cold start emission results (bag emissions) and the limits (standards Euro 4)

Pollutants	Emissions results (g/km)			EU Emission Standards for passenger cars using gasoline engines (g/km)
	Test 1	Test 2	Test 3	
THC	0.046	0.046	0.051	0.1
NMHC	0.041	0.041	0.046	–
CH_4_	0.0047	0.0048	0.0049	–
NO_X_	0.02	0.019	0.016	0.08
CO	0.439	0.441	0.448	1.0
CO_2_	201.411	202.796	203.332	–

**Table 6:** Hot start emission results (bag emissions)

Pollutants	Emissions results (g/km)		
	Test 4	Test 5	Test 6
THC	0.0055	0.0056	0.0085
NMHC	0.0028	0.0026	0.0051
CH_4_	0.0028	0.0032	0.0034
NO_X_	0.0057	0.0023	0.0086
CO	0.15	0.16	0.19
CO_2_	185.4	186.5	186.9

**Fig. 4:**
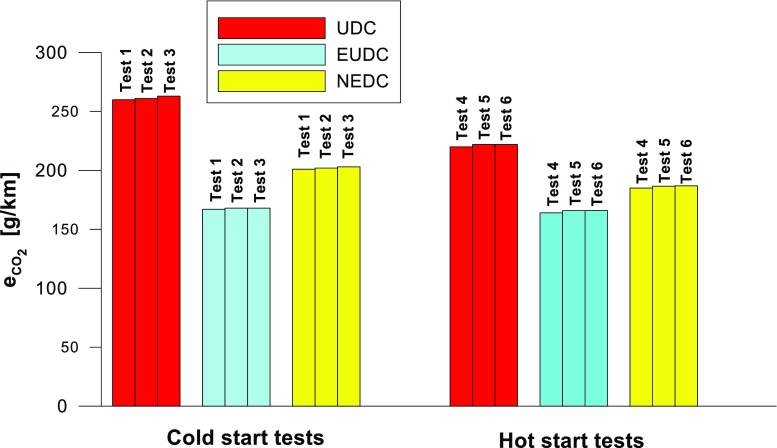
Results of the CO_2_ emission obtained for three tests (from a cold start and from a hot start)

**Fig. 5:**
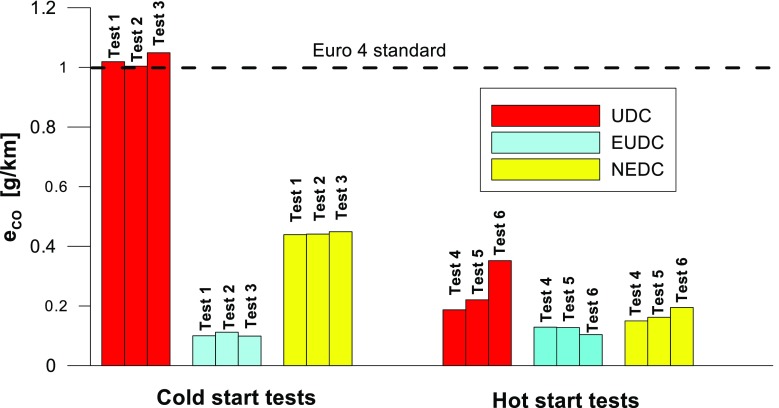
Results of the CO emission obtained for three tests (from a cold start and from a hot start)

**Fig. 6:**
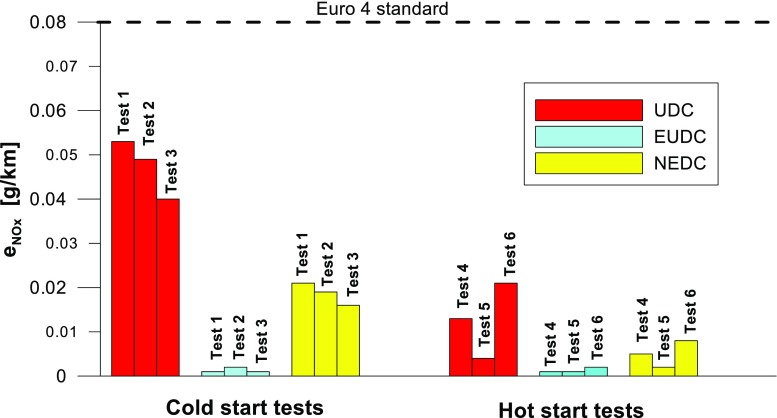
Results of the NO_X_ emission obtained for three tests (from a cold start and from a hot start)

**Fig. 7:**
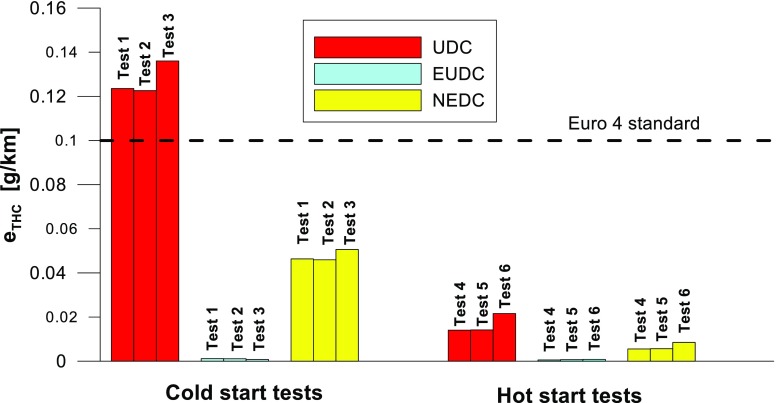
Results of the THC emission obtained for three tests (from a cold start and from a hot start)

**Fig. 8:**
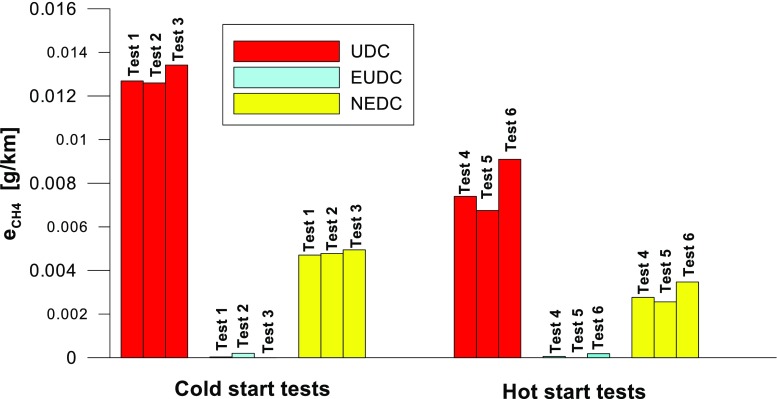
Results of the CH_4_ emission obtained for three tests (from a cold start and from a hot start)

**Fig. 9:**
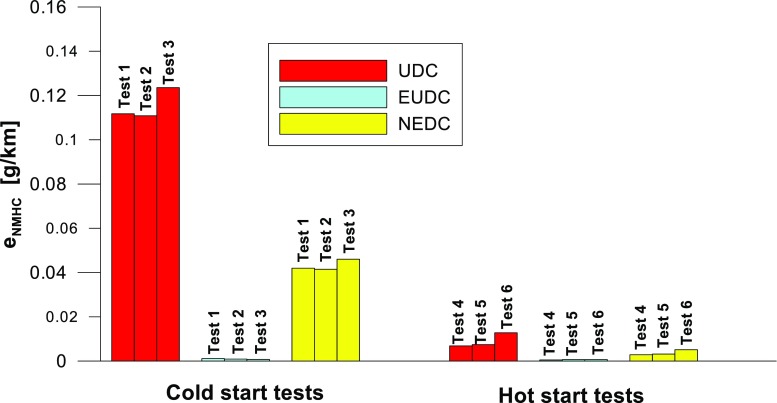
Results of the NMHC emission obtained for three tests (from a cold start and from a hot start)

Figures 4, 5, 6, 7, 8, and 9 compare the emission results of the analyzed pollutants in the exhaust for phase 1 (UDC), phase 2 (EUDC), and the entire test (NEDC).

The values of emission factors of the tested exhaust components are considerably higher in the urban part (UDC). In the case of the EUDC cycle, the emission factors were similar to those from cold and hot starts. This is due to the heating of the engine during the test (especially in the UDC phase during the cold start test). CO_2_ emission (Fig. 4) in the urban phase (UDC) is higher than in the EUDC emission, which is related to, e.g. higher average speed in the EUDC phase. By analyzing the graphs in Figs. 5 and 6, it can be seen that the CO and NO_X_ emission values in the UDC part, in the hot start tests (tests 4–6), differ to a greater extent than in the cold start tests (tests 1–3). This may be due to the engine control strategy and the catalytic reactor efficiency. The results of the test included in paper (Favez et al. 2009) prove that in the examined cars the CO and NO_X_ emissions from the warm engine may, in some phases of the test cycle, be higher than the emissions from the cold start test. According to the authors, this may be connected with the adopted engine control strategy.

However, as has been demonstrated in paper (Shamim 2011), the increase of the exhaust temperature is increased by the difference in the degree of conversion of toxic components (NO_X_, CO, HC) in the catalytic converter, depending on the air/fuel ratio. For lean mixtures (*λ* > 1), the average conversion efficiency of the NO for exhaust temperature 100 °C constituted ≈ 99%, while for an exhaust temperature of 300 °C it was ≈ 6%. In the case of CO and HC, a low conversion efficiency of the catalytic converter occurs for the rich mixtures. The lower exhaust temperature, regardless of the composition of the blend, caused less variation in conversion efficiency of the catalytic converter (Shamim 2011).

With respect to normative emission levels (Euro 4), it is apparent that for all toxic components, i.e. CO (Fig. 5), NO_X_ (Fig. 6), and THC (Fig. 7), the requirements are fulfilled. CH_4_ (Fig. 8) emission has the lowest value among the analyzed components.

**Fig. 10:**
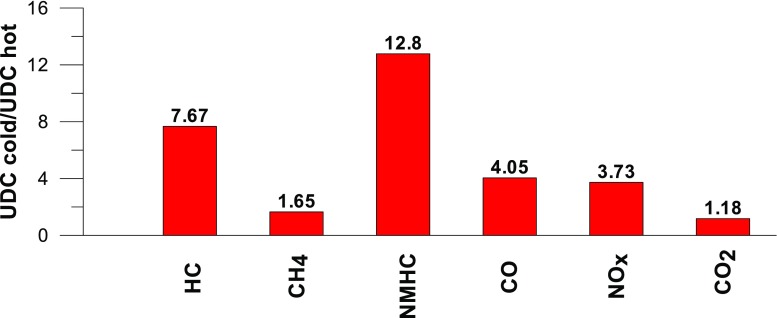
The ratio of the average factor of emission from cold and hot starts for the UDC phase (average of three tests)

**Fig. 11:**
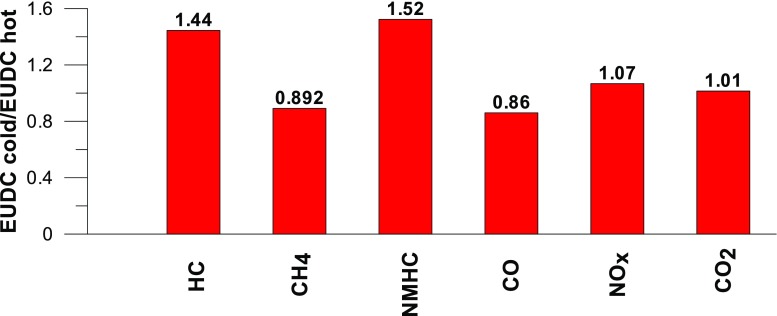
The ratio of the average factor of emission from cold and hot starts for the EUDC phase (average of three tests)

**Fig. 12:**
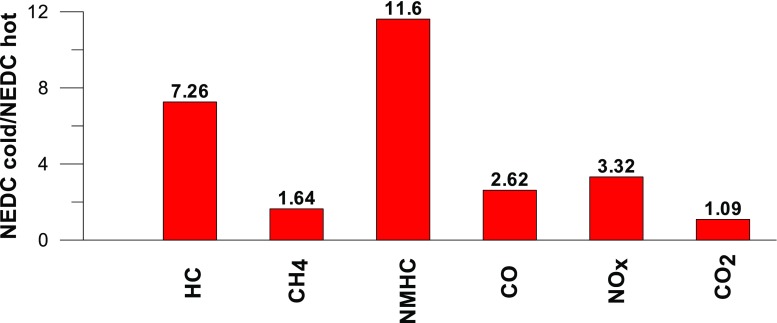
The ratio of the average factor of emission from cold and hot starts for the NEDC cycle (average of three tests)

Taking into account the values of the emission factors of exhaust components analyzed for cold and hot start tests (Figs. 10, 11, and 12), it is apparent that, ignoring the emission of CO and CH_4_ in the second phase (for the EUDC cycle), lower emission for the hot start tests was obtained. For the NEDC tests performed for the hot start (Fig. 12), THC emission was approx. 7 times lower, and in the case of NMHC more than 11 times lower, than in the cold start tests. NO_X_ emission was lower by more than 3 times, while CO_2_ emission was lower by ≈ 9%. This is mainly related to the emission values obtained in the first phase of the cycle—UDC (Fig. 10), for which the differences in the exhaust pollutants emissions are the greatest. For the EUDC cycle (Fig. 11), the differences in the emissions factors from the cold and hot starts were small, which was largely due to the approximate thermal state of the engine being independent of the initial engine test temperatures. The analysis of temperature changes during the NEDC test conducted in the climate chamber (*T* = 22 °C), presented by the authors’ paper (Torregrosa et al. 2008), showed that the engine of the tested car reached the oil temperature of ≈ 80 °C at the end of the UDC phase. Similar results are presented in paper (Suarez-Bertoa et al. 2014). Thus, for the EUDC cycle, the engine’s thermal state is similar for both cold start and hot start tests.

**Fig. 13:**
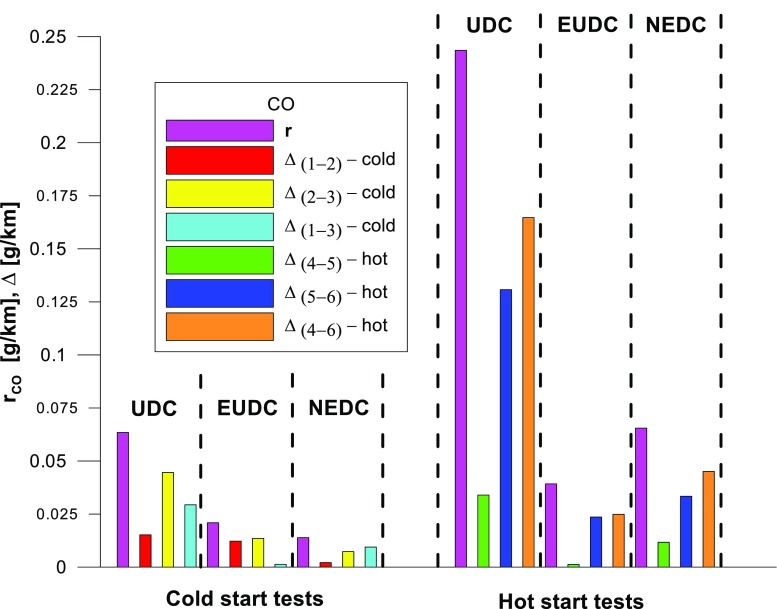
Results of the repeatability limits, *r*, and absolute differences, Δ, for the two emission results of CO from the cold and hot start tests

**Fig. 14:**
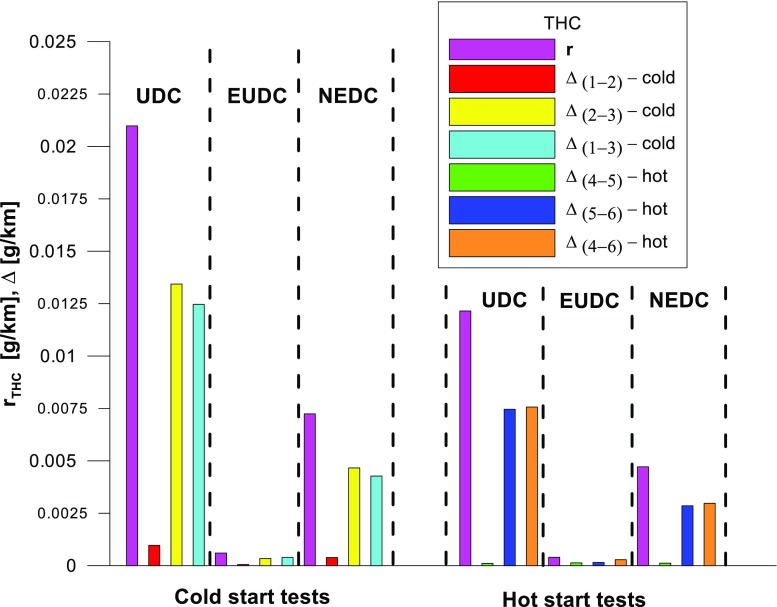
Results of the repeatability limits, *r*, and absolute differences, Δ, for the two emission measurements of THC from the cold and hot start tests

**Fig. 15:**
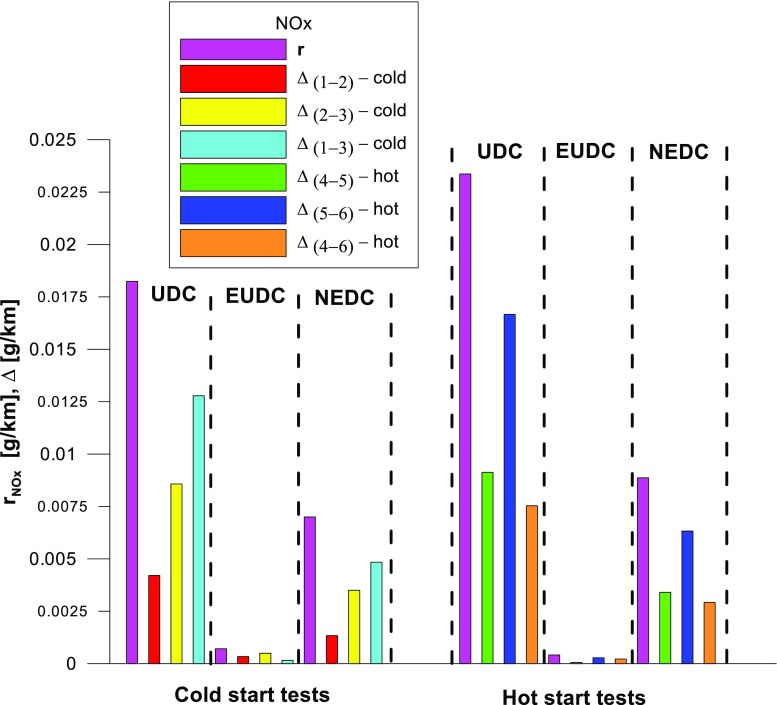
Results of the repeatability limits, *r*, and absolute differences, Δ, for the two emission measurements of NO_X_ from the cold and hot start tests

**Fig. 16:**
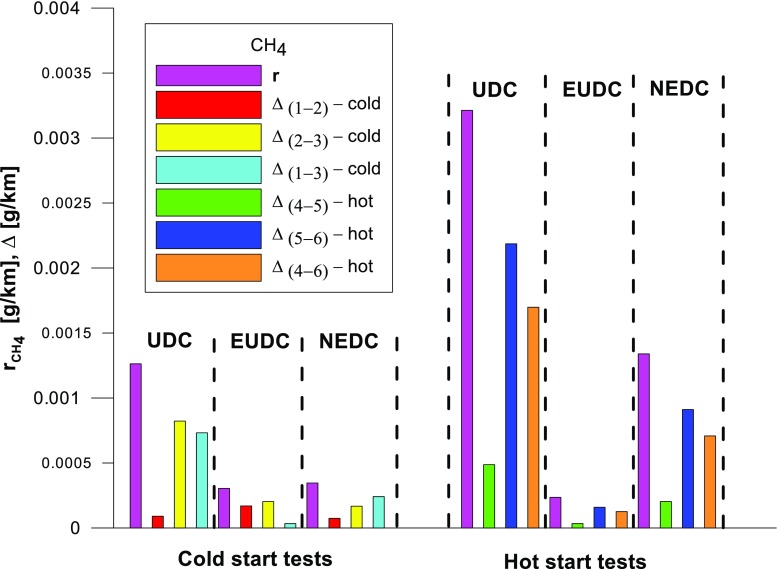
Results of the repeatability limits, *r*, and absolute differences, Δ, for the two emission measurements of CH_4_ from the cold and hot start tests

**Fig. 17:**
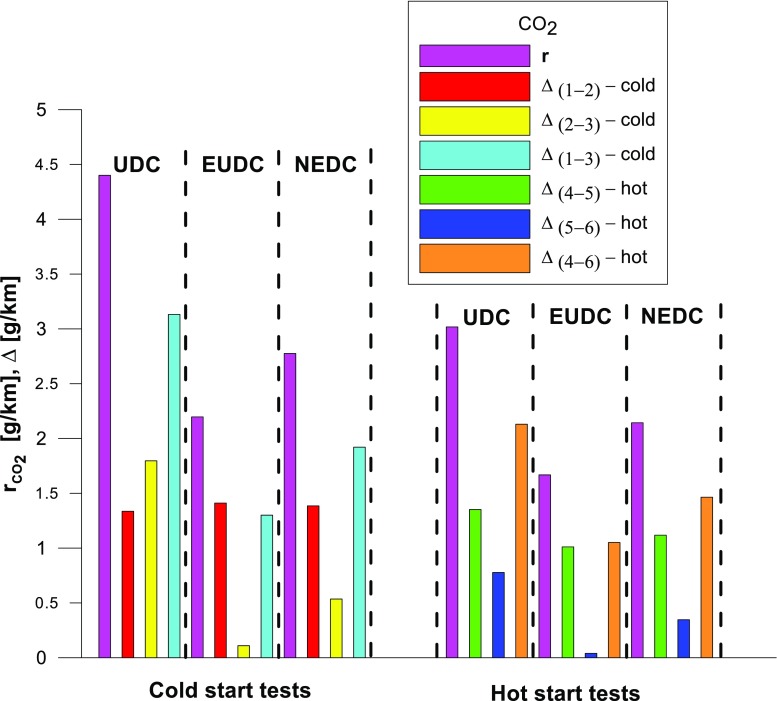
Results of the repeatability limits, *r*, and absolute differences, Δ, for the two emission measurements of CO_2_ from the cold and hot start tests

**Fig. 18:**
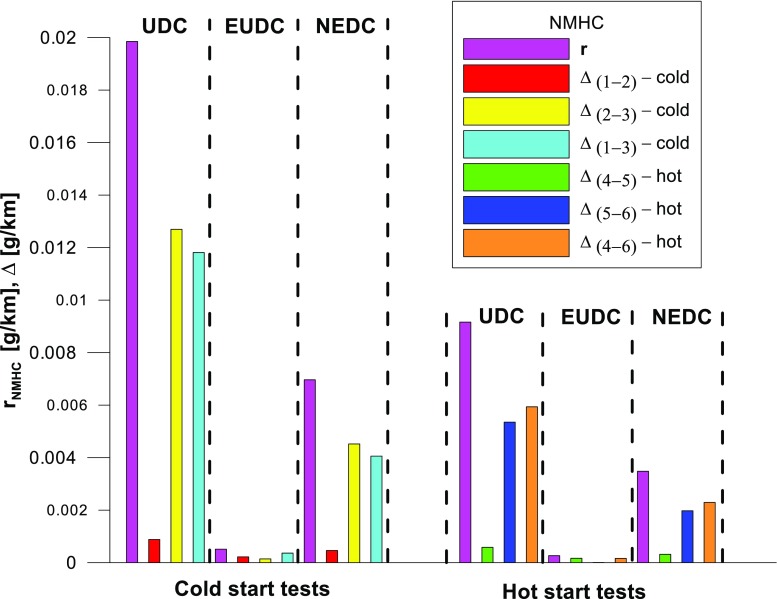
Results of the repeatability limits, *r*, and absolute differences, Δ, for the two emission measurements of NMHC from the cold and hot start tests

**Fig. 19:**
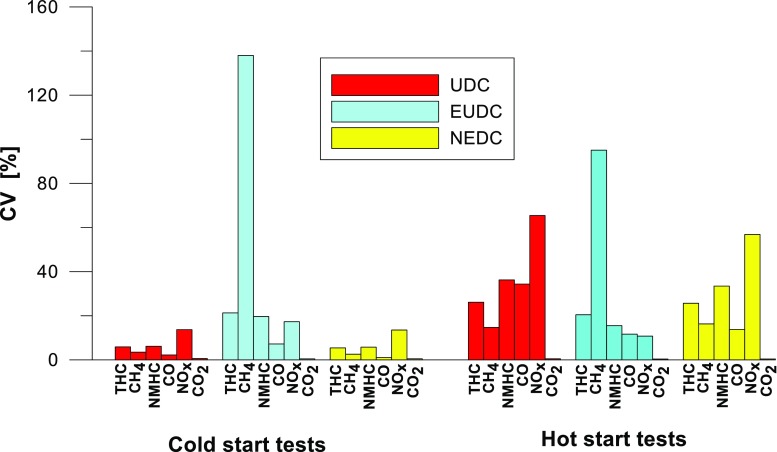
The values of the CV of the analyzed exhaust components for both of the phases and for the entire test

The values of the calculated repeatability parameters for the NEDC test results from the cold start are set out in Table 7, and from the hot start in Table 8. Table 9 lists the allowable differences, Δ, between the results of two emission measurements under repeatability conditions for the analyzed exhaust gas components. Figs. 13, 14, 15, 16, 17, and 18 show the results of the repeatability limits, *r*, and absolute differences for the two emission measurements of the analyzed exhaust components. Figure 19 shows the values of the CV of the specific emission measurements of the analyzed exhaust components for each phase and for the entire test.

**Table 7:** Parameters of repeatability analysis (NEDC—cold start tests)

Parameter	Values for THC	Values for CH_4_	Values for NMHC	Values for NO_X_	Values for CO	Values for CO_2_
*x*_av_ (g/km)	0.048	0.005	0.043	0.018	0.443	202.513
*x*_min_ (g/km)	0.046	0.005	0.041	0.016	0.439	201.411
*x*_max_ (g/km)	0.051	0.005	0.046	0.021	0.449	203.331
*R*_*x*_ (g/km)	0.005	0.0002	0.005	0.005	0.009	1.921
*s*_r_ (g/km)	0.00258	0.00012	0.0024	0.0025	0.0049	0.99
CV (%)	5.43	2.56	5.76	13.53	1.11	0.489
*r* (g/km)	0.0072	0.0003	0.0069	0.007	0.0138	2.7758

**Table 8:** Parameters of repeatability analysis (NEDC—hot start tests)

Parameter	Values for THC	Values for CH_4_	Values for NMHC	Values for NO_X_	Values for CO	Values for CO_2_
*x*_av_ (g/km)	0.007	0.003	0.004	0.006	0.169	186.29
*x*_min_ (g/km)	0.006	0.002	0.003	0.002	0.15	185.43
*x*_max_ (g/km)	0.009	0.003	0.005	0.009	0.195	186.895
*R*_*x*_ (g/km)	0.003	0.001	0.002	0.006	0.045	1.465
*s*_r_ (g/km)	0.00168	0.0005	0.0012	0.0031	0.0234	0.765
CV (%)	25.66	16.31	33.45	56.91	13.82	0.41
*r* (g/km)	0.0047	0.0013	0.0035	0.0088	0.0655	2.1436

**Table 9:** Allowable differences, Δ, between the results of two emission measurements under repeatability conditions for the analyzed exhaust gas components

Exhaust component	Allowable difference Δ	
	Cold start test	Hot start test
THC	Δ ≤ 0.0072 g/km	Δ ≤ 0.0047 g/km
NMHC	Δ ≤ 0.0069 g/km	Δ ≤ 0.0035 g/km
CH_4_	Δ ≤ 0.0003 g/km	Δ ≤ 0.0013 g/km
NO_X_	Δ ≤ 0.007 g/km	Δ ≤ 0.0088 g/km
CO	Δ ≤ 0.0138 g/km	Δ ≤ 0.0655 g/km
CO_2_	Δ ≤ 2.7758 g/km	Δ ≤ 2.1436 g/km

The *r* values of the CO and NO_X_ emissions for the tested car were higher for the UDC phase of the NEDC tests, implemented from the hot start in relation to the results obtained from the cold start (Figs. 13 and 15).

For cold start tests (Table 7), the highest value of *s*_r_ for the entire NEDC test was obtained for the emissions of CO_2_ with the value of 0.99 g/km, and the lowest value *s*_r_ = 0.0012 g/km for CH_4_. For the remaining measured exhaust components, the standard deviation values were small and amounted to ≈ 0.0025 g/km for NO_X_, NMHC, and THC. In the case of CO emission, the absolute standard deviation was ≈ 0.0049 g/km for the cold start tests.

For hot start tests (Table 8), the highest value of *s*_r_ for the entire NEDC test was, as before, obtained for the emissions of CO_2_ with the value of 0.765 g/km, and the lowest value *s*_r_ = 0.0005 g/km for CH_4_. For THC and NMHC, the standard deviation values were lower than those from a cold start and were respectively ≈ 0.0017 and ≈ 0.0012 g/km. With regard to the results of the NO_X_ emission, the standard deviation from the hot start test was greater than for the cold start tests (by ≈ 25%) and amounted to 0.0031 g/km. A significant value of the standard deviation for CO emissions of ≈ 0.023 g/km was obtained in the case of hot start tests. This value was 4.5 times greater than the value obtained for cold start tests, which may be related to the abovementioned engine control strategy and conversion efficiency of the catalytic reactor. Similar results were obtained by the authors of paper (Clairotte et al. 2013), where for a passenger car (Euro 4) fueled with E5 in the NEDC cold start test (at 22 °C), the following values of standard deviations were obtained: 0.007 g/km for HC, 0.007 g/km for NMHC, 0.127 g/km for CO, 0.003 g/km for NO_X_, and 0.9 g/km for CO_2_.

Values of *r* for the NEDC cycle during hot start tests (Table 8), were higher for CH_4_, NO_X_, and CO (respectively: 0.0013 g/km, 0.0088 g/km, 0.0655 g/km) in relation to those measures obtained from the cold start (0.0003 g/km, 0.007 g/km, 0.0138 g/km). For the remaining exhaust components analyzed, the repeatability limit for cold start tests was higher than for hot start tests (by ≈ 53% for THC, by ≈ 97% for NMHC, and by ≈ 29% for CO_2_).

The highest values of repeatability limits were obtained for the analyzed pollutants in the urban part of the UDC, where a greater variability of the test results occurs. In hot start tests for CO (Fig. 13), CH_4_ (Fig. 16), and NO_X_ (Fig. 15), the value of this parameter was higher than in the cold start tests. This may be related to the mentioned engine control strategy.

CV values (Fig. 19) for the hot start tests were higher than those obtained from the cold start (in the cold start tests, the CV did not exceed 10%, except for NO_X_). The lowest CV values were obtained for specific emission of CO_2_, both for the entire test, as well as for each of its phases (the maximum value of this parameter was ≈ 0.6%). Low variability was also evident in the specific emission of CO (CV for the entire cold start test was ≈ 1%, while for the hot start was ≈ 14%). The highest CV values were obtained for specific emission of CH_4_ (≈ 138% in the EUDC cycle from the cold start and ≈ 95% in the EUDC cycle from the hot start). High variability was also obtained for specific emission results of NO_X_ for the hot start tests in the UDC cycle (≈ 65%). For cold start tests, a high CV value (≈ 21%) was obtained for specific emission of THC in the EUDC cycle. For the entire NEDC cold start, the highest variability (CV = 13%) was observed in the specific emission of NO_X_. Similarly, for the hot start for the entire NEDC test, the greatest variation was obtained for NO_X_ (CV = 57%). It should be borne in mind that the values of the CVs depend on the absolute values of the pollutants emissions measured, hence their values depend on the accuracy of the measuring equipment used, which is of particular importance for the exhaust components for which the absolute values of the emissions are low.

## Conclusions

The paper presents research on the accuracy of the results for exhaust pollutants emission, conducted under repeatability conditions for a small number of tests. For this purpose, a passenger car with Euro 4 homologation was used; moreover, the results of measurements proved that despite the mileage it still met the Euro 4 emission limits. The analysis included tests performed in the climate chamber according to the NEDC test both from cold (coolant temperature of 21 ± 1 °C) and hot (coolant temperature of 90 ± 2 °C) starts. For the measured exhaust components (CO, THC, CH_4_, NMHC, NO_X_, CO_2_), the accuracy parameters were indicated for the first phase of the test (UDC), the second phase of the test (EUDC), and for the entire test (NEDC).

The conducted research allows us to formulate the following conclusions:

- A proper interpretation of the results of the pollutant emission tests based on the mean value, in particular for a small number of tests, requires the results repeatability analysis.
- A temperature of the engine at the test start do not affect significantly the emission values recorded in phase 2 of the cycle (EUDC).
- A higher engine temperature at the test start is associated with greater variability (CV) of the results obtained for the NEDC cycle.
- The results obtained in phase 1 (UDC) have the greatest impact on the results of the emission for the entire test.
- A higher engine temperature at the test start is associated with lower values of repeatability r for the NEDC cycle and CO, CH_4_, and NO_X_ emissions.
- For a more complete assessment of the pollutant emission results obtained for a small number of measurements, i.e., when they may be subjected to a large scattering, it is advantageous, according to the authors, to determine the parameter allowing the evaluation of repeatability of obtained values. The use of that coefficient can be helpful in the evaluation of the repeatability of the results obtained by various laboratories.
- The coefficient of variation (CV) that is used in such cases for assessing variability is unsuitable when very low emission values are recorded. However, such achievements are desirable from environment protection point of view.
- Since the CV values depend on their average values, the authors suggest to define the threshold coefficient of variation (CVL) determined relatively to the standard emission limit value of a given exhaust compound (L), instead of the average value. The values of the proposed CVL do not depend on the mean values, so they can be used for evaluation of repeatability of the test results.
- Currently, there are no satisfactory standards specifying the CV values relating to the results of pollutant emission obtained during steady-state driving cycles. In the case of the standard of Euro 4, which was implemented for the tested vehicle, the maximum threshold CVL should be less than 20%.
- For the conducted cold start tests, the values of the defined here CVL for THC, CO, and NO_X_ emissions are respectively equal to 2.6, 0.5, and 3.1%. The obtained values of CVL for harmful exhaust compound emission results (CO, THC, NO_X_) are acceptable for cold start tests.

Based on the analysis of obtained test results, authors conclude that the performing procedures for driving cycles have been implemented correctly. The steps taken to establish the satisfactory values of the coefficients of variation separately for each analyzed exhaust component appear to be desired. The research carried out by the authors show that the selection of acceptable values of this coefficient should consider the thermal state of the engine at test start. This objective requires a statistical series of results obtained from various laboratories.

## Glossary

CH_4_: Methane
CO: Carbon monoxide
CO_2_: Carbon dioxide
CV: Coefficient of variation
CV_L_: Threshold coefficient of variation
CVS: Constant volume sampling
EUDC: Extra Urban Driving Cycle
NEDC: New European Driving Cycle
NMHC: Non-methane hydrocarbons
NOx: Nitrogen oxides
*r*: Repeatability limit
R&D: Research and development
RDE: Real Driving Emissions
R_x_: Range (*x*_max_-*x*_min_)
*S*_r_: Repeatability standard deviation
THC: Total hydrocarbons
UDC: Urban Driving Cycle
WLTC: World Harmonized Light Vehicles Test Cycle
WLTP: Worldwide Harmonized Light Vehicles Test Procedure
*x*_av_: Mean value
*x*_max_: Maximum value
*x*_min_: Minimum value
*Δ*: Allowable differences, between the results of two emission measurements under repeatability conditions
*λ*: Relative air-fuel ratio
